# Supplementary material for: Global Methylome Scores Correlate with Histological Subtypes of Colorectal Carcinoma and Show Different Associations with Common Clinical and Molecular Features
Source: Cancers (Basel). 2021 Oct 14;13(20):5165. doi: 10.3390/cancers13205165 (PMC8533997; doi:10.3390/cancers13205165)
Supplement: Supplementary file 1 [file cancers-13-05165-s001.zip › cancers-1363764-supplementary.pdf]

Global Methylome Scores Correlate with Histological Subtypes of Colorectal Carcinoma and Show Different Associations with Common Clinical and Molecular Features

María del Carmen Turpín-Sevilla, Fernando Pérez-Sanz, José García-Solano, Patricia Sebastián-León, Javier Trujillo-Santos, Pablo Carbonell, Eduardo Estrada, Anne Tuomisto, Irene Herruzo, Lochlan J. Fennell, Markus J. Mäkinen, Edith Rodríguez-Braun, Vicki L. J. Whitehall, Ana Conesa and Pablo Conesa-Zamora

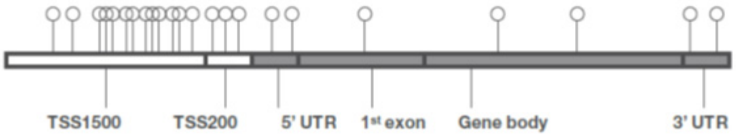

Feature Type	Genes Mapped	Percent Genes Covered	Number of Loci on Array
NM_TSS200	14895	0.79	2.56
NM_TS1500	17820	0.94	3.41
NM_5'UTR	13865	0.78	3.34
NM_1stExon	15127	0.80	1.62
NM_3'UTR	13042	0.72	1.02
NM_GeneBody	17071	0.97	8.97
NR_TSS200	1967	0.65	1.84
NR_TSS1500	2672	0.88	2.92
NR_GeneBody	2345	0.77	5.34

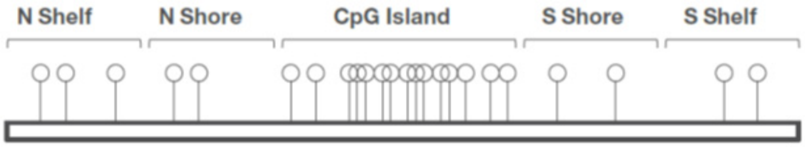

Feature Type	Islands Mapped	Percent Islands Covered	Average Number of Loci on Array
Island	26153	0.94	5.08
N_Shore	25770	0.93	2.74
S_Shore	25614	0.92	2.66
N_Shelf	23896	0.86	1.97
S_Shelf	23968	0.86	1.94

Figure S1. These regions were defined based on the manifest and annotation data from the R-packages "IlluminaHumanMethylation450kmanifest" [25] and "IlluminaHumanMethylation450kanno.ilmn12.hg19" [36].

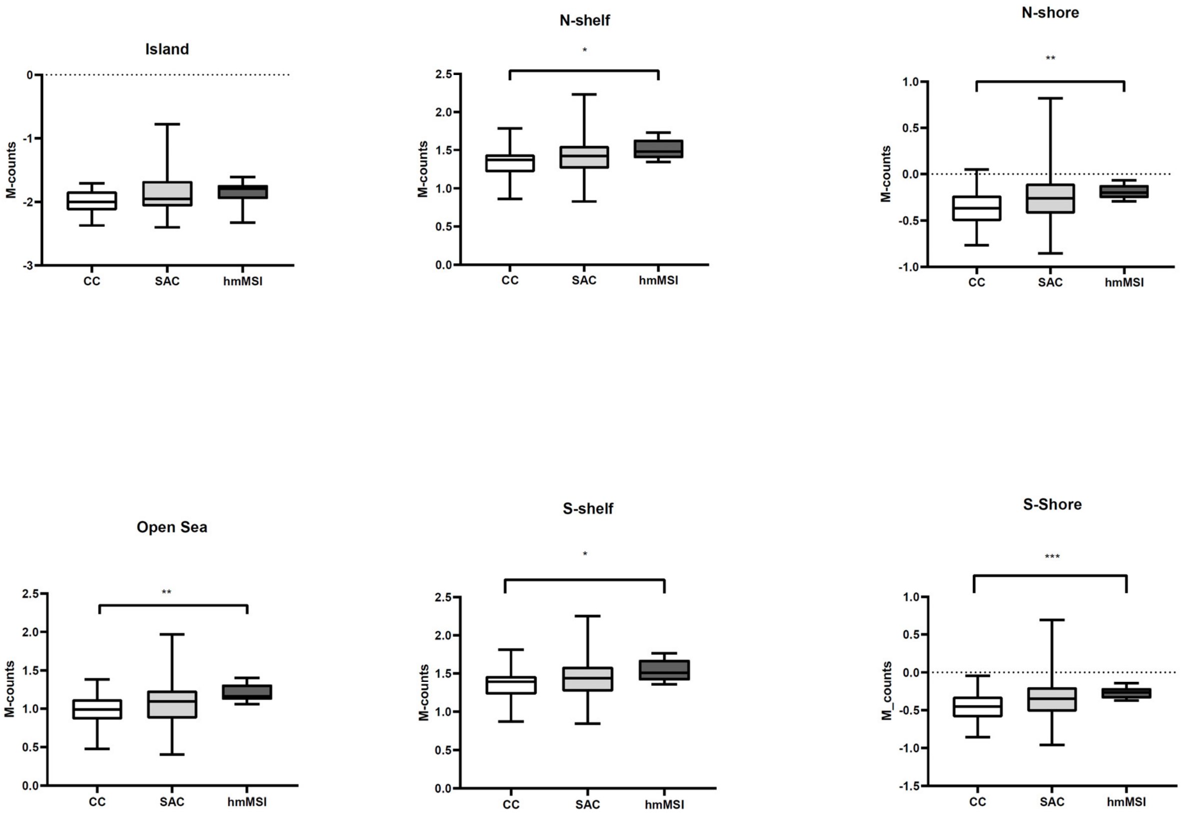

Figure S2. Boxplot graphs showing the methylation differences in the regions referred to CpG islands amongst the different histological CRC subtypes. * $p < 0.05$, ** $p < 0.01$, *** $p < 0.001$.

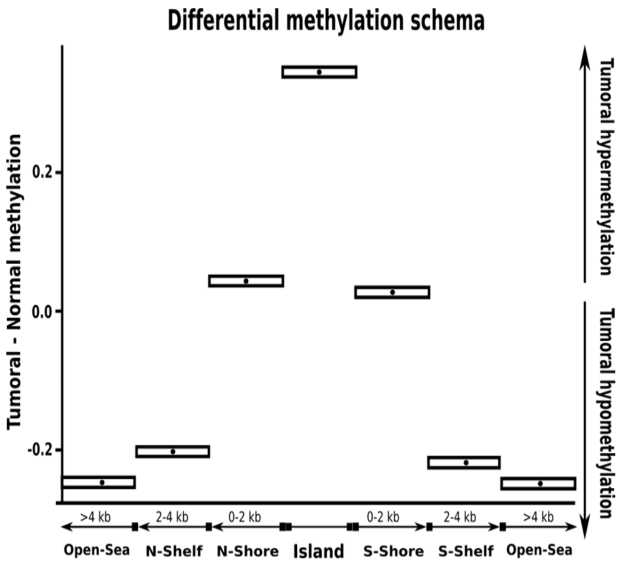

Figure S3. Differential methylation schema (tumoral CRC M-value–normal adjacent colorectal mucosa M-value) showing the tumor-associated hyper- and hypomethylation according to the genome locations with respect to the CpG island.

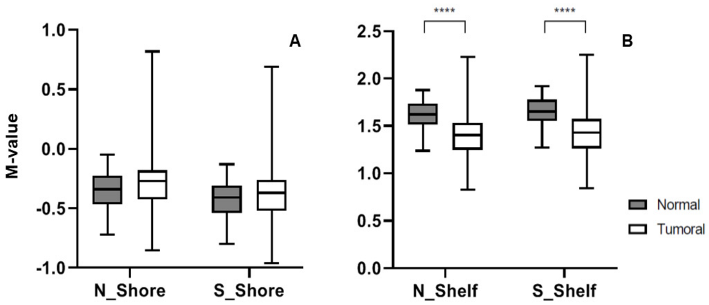

Figure S4. Comparison between normal and tumoral methylation in North and South directions of Shores (**A**) and Shelves (**B**) areas with respect to CpG island. **** $p < 0.0001$.

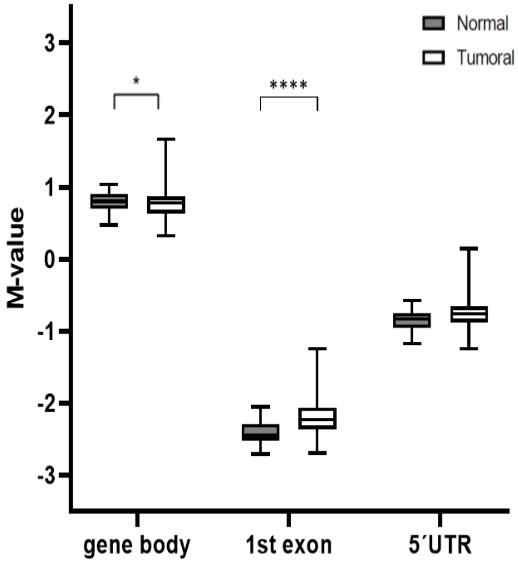

Figure S5. Comparison between normal and tumoral methylation in different gene- related locations; i.e. 5´Untranslated Region (5´UTR), First exon and gene body *$p > 0{,}05$, **** $p < 0.0001$.

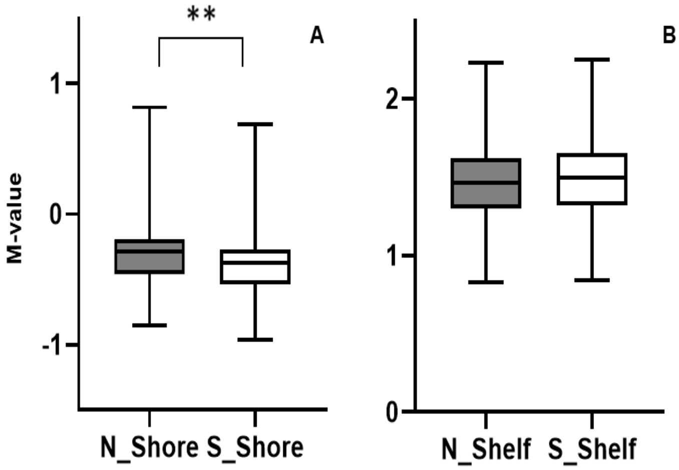

Figure S6. Comparison between normal and tumoral methylation in different locations; North and South directions of Shores (**A**) and Shelves (**B**) areas with respect to CpG island. ** $p < 0.01$

A

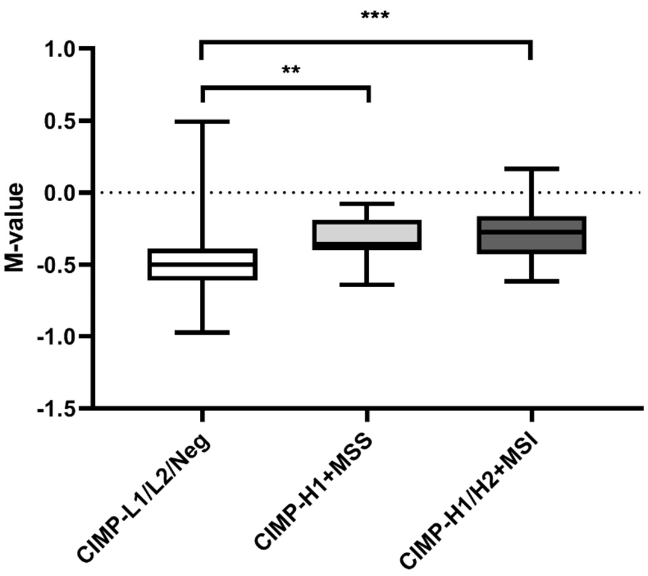

B

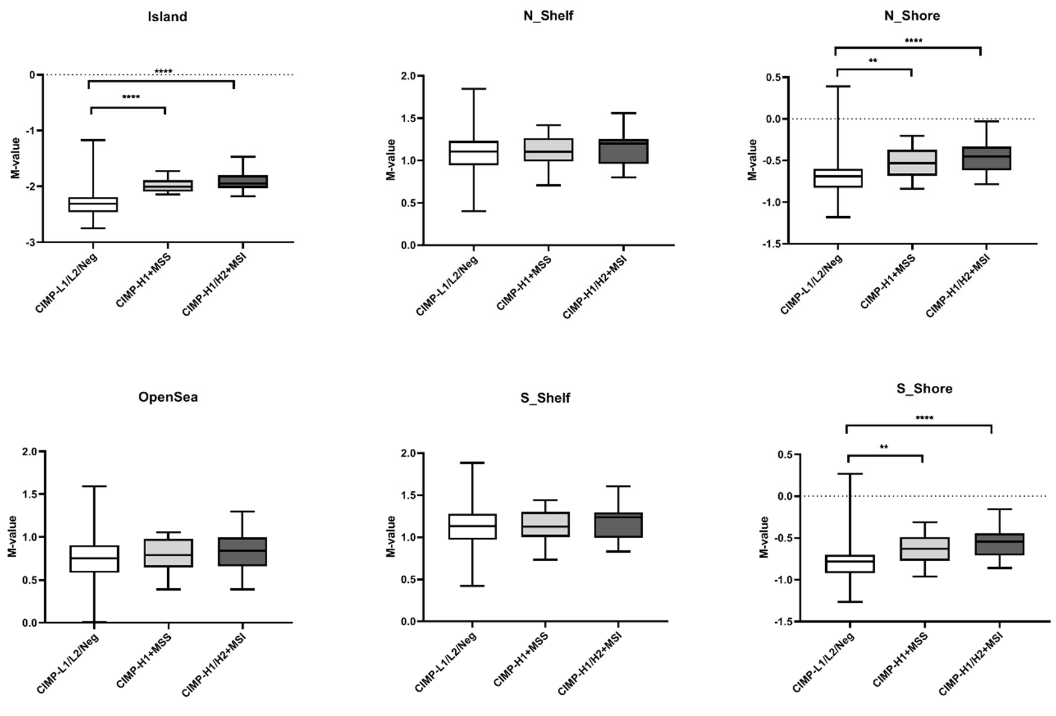

Figure S7. Validation of global methylation patterns in an independent series. (**A**)**,** boxplot graph showing the global methylation level amongst the groups enriched in CC (CIMP-L1-L2-Neg/CIMP-H2/MSS+), SAC (CIMP-H1/MSS+), and hmMSI-H (CIMP-H1-H2/MSI+) histological subtypes. Krustal Wallis test ****$p < 0.0001$. Mann Whitney Test (graphs) ** $p < 0.01$, *** $p < 0.001$ (**B**), boxplot graphs showing the methylation differences in the regions referred to CpG islands amongst the groups enriched in CC (CIMP-L1-L2-Neg/CIMP-H2/MSS+), SAC (CIMP-H1/MSS+), and hmMSI-H (CIMP-H1-H2/MSI+) histological subtypes ** $p < 0.01$, **** $p < 0.0001$.

Table S1. Association of global methylation scores of adjacent normal mucosa specimens with histopathological features.

			250 bp			1 kb			2 kb		
		n	**Median**	*IQR*	*p*	**Median**	**IQR**	*p*	**Median**	**IQR**	*p*
Status	normal	35	0.2660	0.0034	0.0100 *	0.4224	0.0083	<0.0001 *	0.6345	0.0157	<0.0001 *
	tumoral	82	0.2698	0.0106		0.4106	0.0211		0.5943	0.0309	
Type	CC										
	normal	14	0.2664	0.0033	0.8110	0.4232	0.0054	<0.0001 *	0.6358	0.0077	<0.0001 *
	tumoral	32	0.2653	0.0092		0.4051	0.0198		0.5871	0.0271	
	SAC										
	normal	15	0.2662	0.0035	0.0160	0.4222	0.0113	0.0010 *	0.6360	0.0274	<0.0001 *
	tumoral	40	0.2705	0.0104		0.4121	0.0205		0.5933	0.0341	
	MSI										
	normal	6	0.2646	0.0073	0.0050 *	0.4208	0.0102	0.5620	0.6272	0.0222	0.0930
	tumoral	10	0.2764	0.0095		0.4237	0.0142		0.6132	0.0347	

Note: *p*-values for Mann-Whitney's *U* test. Chi-squared tests applied for testing the bivariate associations of each variable with type of tumor. The Bonferroni correction for multiple comparisons was applied. Associations with type of tumor are considered significant (marked with *) if $p < 0.05/4 = 0.0125$. CC: Conventional Carcinoma, SAC: Serrated adenocarcinoma, hmMSI-H: Colorectal cancer showing histological and molecular features of high level of microsatellite instability.

Table S2. Associations between global methylation scores with the methylation status of the genes included in the CIMP panel.

			250 bp			1 kb			2 kb		
		n	**Median**	**IQR**	*p*	**Median**	**IQR**	*p*	**Median**	**IQR**	*p*
**CAGNA1G**	**Unmet**	55	0.2681	0.0105	0.0530	0.4079	0.0210	0.2430	0.5902	0.0316	0.3640
	**Met**	25	0.2721	0.0115		0.4152	0.0219		0.5974	0.0301	
**CDKN2A**	**Unmet**	39	0.2665	0.0084	0.0110	0.4066	0.0219	0.5220	0.5900	0.0276	0.7250
	**Met**	41	0.2721	0.0109		0.4152	0.0236		0.5970	0.0334	
**CRABP1**	**Unmet**	39	0.2676	0.0098	0.1270	0.4095	0.0225	0.9190	0.5939	0.0334	0.6000
	**Met**	41	0.2709	0.0128		0.4115	0.0220		0.5939	0.0301	
**IGF2**	**Unmet**	14	0.2656	0.0089	0.1840	0.4093	0.0245	0.9600	0.5981	0.0408	0.5020
	**Met**	66	0.2702	0.0115		0.4114	0.0210		0.5939	0.0281	
**MLH1**	**Unmet**	59	0.2676	0.0101	0.0260	0.4079	0.0214	0.2110	0.5900	0.0313	0.4600
	**Met**	21	0.2731	0.0104		0.4152	0.0197		0.5974	0.0281	
**NEUROG1**	**Unmet**	22	0.2667	0.0096	0.1060	0.4093	0.0238	0.8800	0.5952	0.0327	0.6130
	**Met**	58	0.2702	0.0114		0.4122	0.0210		0.5933	0.0309	
**RUNX3**	**Unmet**	48	0.2686	0.0100	0.1300	0.4085	0.0222	0.8440	0.5939	0.0319	0.6510
	**Met**	32	0.2721	0.0143		0.4135	0.0211		0.5941	0.0311	
**SOCS1**	**Unmet**	78	0.2698	0.0106	0.4110	0.4106	0.0215	0.7970	0.5943	0.0309	0.2410
	**Met**	2	0.2747			0.4073			0.5747		

Note: *p*-values for Mann-Whitney's *U* test. The Bonferroni correction for multiple comparisons was applied for each site; *p* values $< 0.05/8 = 0.0063$ were considered statistically significant.
